# Psychophysiological Arousal and Auditory Sensitivity in a Cross-Clinical Sample of Autistic and Non-autistic Anxious Adults

**DOI:** 10.3389/fpsyt.2018.00783

**Published:** 2019-01-29

**Authors:** David Nicholas Top Jr., Steven G. Luke, Kevin G. Stephenson, Mikle South

**Affiliations:** ^1^Department of Psychology, Brigham Young University, Provo, UT, United States; ^2^Neuroscience Center, Brigham Young University Provo, UT, United States; ^3^Nationwide Children's Hospital, Columbus, OH, United States

**Keywords:** autism spectrum disorder, sensory processing, anxiety, intolerance of uncertainty, anxious arousal, pupillometry, habituation

## Abstract

Many autistic people report overwhelming sensory experiences and also elevated levels of anxiety. Understanding how these experiences are linked to each other can contribute to improved support and intervention for reducing sensory overload and anxiety. This study included 95 young adult participants including autistic adults, non-autistic adults reporting to a psychotherapy clinic with high levels of anxiety, and neurotypical adults with no psychiatric concerns. We measured pupil size using including a baseline task with no auditory stimulus followed by two blocks of simple auditory habituation. In a subset of 80 participants we also measured self-report levels of sensory processing, anxious apprehension, and intolerance of uncertainty. The autism group showed atypical sensory processing on all four measured domains of the Adolescent and Adult Sensory Profile including sensory sensitivity, sensory seeking, sensory avoidance, and low registration subscales. Dimensional analyses across all participants showed significant positive correlations between sensory sensitivity, sensory seeking, and sensory avoidance domains with scores from the Intolerance of Uncertainty Scale-Short Form and Penn State Worry Questionnaire. The autism group showed significantly larger pupil size than other groups at baseline, before any auditory stimulation. There were no group differences in the rate of auditory habituation, nonetheless the overall, absolute larger pupil size remained in the autism group throughout the experiment. We suggest that this and other findings could indicate chronic hyperarousal in many autistic people. Treatment for anxiety in autism should be informed by knowledge of unique aspects of anxiety in autism and consider the role of sensory experience and everyday psychophysiological arousal.

## Introduction

Many autistic people report unusually intense sensory processing, including hypersensitivity to multiple sensory modalities and high levels of distress even to low-threshold sensory stimuli (1, 2). Atypical sensory processing has been reported in between 45 and 95% of autistic samples (3–5) and is included in the most recent definitions of autism (6, 7). Many autistic people also experience elevated levels of anxiety (8–10). These anxiety symptoms can cause significant additional distress, and have been linked to increased levels of problematic behavior (11, 12), difficulty with decision making (13), and considerable stress on family systems (14). While some autistic individuals manifest anxiety in ways typical of other anxious people, there are also unexpected expressions of anxiety in autism that may go overlooked, such as different underlying drives toward compulsive behavior or social avoidance than typically seen in anxiety (9).

There is growing evidence for a strong link between atypical sensory processing and elevated anxiety in autism (15–20). Effective sensory processing is a critical evolutionary component for managing stress and danger [see 21–23] and there are demonstrated links between sensory sensitivity and affective disorders, including anxiety, outside of autism (24–27). However, side-by-side comparisons of young autistic and neurotypical children suggest that the relationship between sensory processing and anxiety may be much more prominent in autism than neurotypical development (28). Green et al. (29) found that sensory over-responsivity emerges earlier than anxiety in autism and that sensory sensitivity predicts later anxiety symptoms in autism.

To date, most studies of the relationship between sensory processing, anxiety and autism traits have focused on child samples. One of the few studies of adults featured mothers with autistic children (30). Among these mothers, 98% of the sample had scores at least a standard deviation above the mean on at least one sensory domain. In a study focused on emotion processing (i.e., alexithymia) in autistic adults, Milosavljevic et al. (31) reported self-report data from autistic adults using the Adolescent Adult Sensory Profile [AASP; (32)] that were somewhat above published norms for the AASP. However, the authors did not administer the AASP to comparison groups and did not report analyses of association between sensory processing and anxiety. Thus, the first primary aim of our study was to directly compare sensory processing behaviors, alongside measures of anxious apprehension and autism traits, in a cross-clinical sample of autistic, anxious, and neurotypical adults.

*Intolerance of uncertainty* (IU), a transdiagnostic psychological construct that refers to decreased thresholds for ambiguity and enhanced discomfort with ambiguity (33), has emerged as a critical mediator between sensory processing and anxiety in autism and other anxiety disorders (18–20, 28, 34–36). Although IU is typically a factor associated with generalized anxiety disorder, IU has shown to negatively affect depression as well as other anxiety disorders (37, 38). Because many autistic individuals prefer things to be predictable and dislike change, it has been argued that characteristics of IU share some common features with the insistence on sameness seen in autism (34). Multiple studies have now established the link between IU, anxiety, and ASD symptomology (28, 34, 35, 39). A study by Boulter et al. (34) reported a “causal meditational model” in which IU almost completely mediated the relationship between the diagnostic group and anxiety scores. Another study, using an autism only sample, found a link between sensory over-responsiveness, IU, and anxiety in which IU mediated the relationship between sensory processing and anxiety (20). Neil et al. (28), replicating the (20) study with a larger sample that includes typically developing individuals, found that IU had a direct effect on sensory sensitivity and anxiety. Given the evidence of IU in modulating the anxiety symptoms in autism, we further evaluated associations between sensory processing, anxiety, and IU in this study.

Another characteristic of studies in this area is a reliance on questionnaires including parent-report surveys (16, 17, 20, 34, 40) or self-report surveys (19, 31, 41). There have been a few notable studies involving psychophysiological measures. Corbett et al. (15) reported that cortisol response to stress was higher for autistic children than neurotypical controls, during an ecologically-relevant peer interaction. In that study greater sensory dysfunction was associated with increased stress, and diagnosis was a significant moderator of the relationship between sensory function and stress response. An emerging idea from our lab and the work of others is that everyday psychophysiological arousal may be elevated in autism (35, 42, 43). We do not know of any studies that examine possible links between ambulatory arousal and sensory and/or emotional sensitivity. It is likely that the sensory and performance demands of laboratory settings would exacerbate such links.

With this limition in mind, some studies have found that autism samples have a larger tonic pupil size–indicative of elevated physiological arousal–than neurotypical comparison groups (44, 45) though others have found no difference (46) or the opposite trend (47). Takahashi et al. (42) found an elevated startle response in autistic children to the mild stimuli as well as a longer peak-startle latency, while a different, threat-modulated startle study found elevated startle response during baseline but not during habituation conditions (35). Our second aim was thus to evaluate evidence for elevated arousal in autism. To do this we designed an explicit extended baseline period to measure tonic pupil size without any other task demands, as well as tracked their pupil size thorughout the duration of the task.

The study of habituation may be useful for understanding the link between sensory processing, anxiety and autism especially with regard to amygdala and insula function in the brain (48–50). In experimental work with both mice and humans, Herry et al. (51) have reported that unpredictability in sequences of sound pulses, which disrupts habituation, is associated with anxiety-like behavior, and is further associated with enhanced/sustained amygdala activity in both animal and human models. The authors suggest that uncertainty at initial encoding (including the amygdala) decreases the flexibility of downstream emotional response. Atypical habituation in autism could therefore underlie inflexible and anxious behavior.

Two fMRI studies of cognitively-typical autistic youth (52, 53) have shown that, during a challenge of mildly aversive sensory stimuli, the autism sample showed more activation than controls in primary sensory areas, amygdala, and orbitofrontal cortex. This activation was correlated with parent-reported anxiety and also with sensory over-responsiveness beyond the association with anxiety. Brain activity in the ASD samples was especially heightened when multiple sensory modalities (auditory and tactile) appeared simultaneously. The authors highlighted difficulties with habituation as a possible underlying feature of sensory overresponsiveness. Takahashi et al. (42) did not find differences in habituation between autistic and neurotypical children during a acoustic startle response paradigm, but a number of other studies have shown reduced or atypical habituation (or increased sensitization, which is the opposite of habituation) in autistic children for various stimulus modalities (54–56). Given the limited literature on sensory experience in adults, our third aim was to characterize unimodal sensory habituation in autistic adults, during a simple auditory habituation task while measuring pupil dilation at baseline and then during two sets of trials which increased in stimulus aversiveness. The sample included autistic adults with typical cognitive performance (AUT group) alongside two IQ-matched adult comparison groups: a sample of highly anxious, (ANX group) and a sample of neurotypical adults who reported no psychiatric concerns (NT group). The inclusion of a highly-anxious group allowed for more direct comparison of the relative contributions of sensory traits and physiological arousal vis-à-vis anxiety in autism.

*Aim 1: Evaluate sensory processing behaviors, and their link to measures of anxious apprehension and autism traits, in autistic adults vis-à-vis clinical and non-clinical comparison groups*. We predicted three-tier outcomes where the autism group would score highest (AUT>ANX>NT) on sensory experience, intolerance of uncertainty, autism trait measures, while the ANX group highest on a measure of anxious apprehension (ANX>AUT>NT). Following our previous study that used a dimensional approach to examine trait-based associations (39), we planned to pool all participants for correlation analyses. We predicted strong associations between sensory experience, anxious apprehension, and intolerance of uncertainty. We also conducted follow-up analyses of correlations within each group separately.

*Aim 2: Compare baseline (non-task) physiological arousal and general physiological arousal (whole experiment) across the autism and comparison groups*. We explicitly measured baseline arousal before the start of the habituation protocol used in this study. We predicted increased pupil size at baseline in the AUT group compared to neurotypical controls. Given previous mixed literature we did not have a firm prediction on whether the AUT group might be equal to or exceed baseline arousal compared to the ANX group. We also predicted a three-tier difference in general arousal throughout the duration of the experiment (ASD <ANX <CON), meaning that the ASD group's general arousal would decrease over time less than the other groups.

*Aim 3: Evaluate sensory habituation in an auditory stimulation task using pupillometry to index psychophysiological arousal*. For this aim we also predicted a three-tier habituation response (AUT<ANX< NT), meaning that pupil dilation would take longest to decrease over each set of trials in the AUT group.

## Materials and Methods

### Participants

Pupillometry data were collected from 95 young adults including 31 AUT group (24 males), 28 ANX group (11 males) and 36 NT group (22 males) participants. A subset of this sample completed the Adolescent Adult Sensory Profile and other behavioral measures (AUT *n* = 24, ANX *n* = 20, NT *n* = 36).

The majority of participants in the AUT group were recruited from a pre-existing database of persons who had participated in previous studies and consented to be contacted for future. Other AUT participants were recruited from the community via recruitment fliers as approved by the Brigham Young University Institutional Review Board. Members in the AUT group had a confirmed diagnosis of autism spectrum disorder informed by the Autism Diagnostic Observation Schedule, Second Edition [ADOS-2; (57)] administered by a research reliable clinician who was also an author of this study.

The ANX group was recruited from individuals with no reported history of autism, who were presenting for psychotherapy at a counseling center of a large private university and had not yet begun, or only just begun psychotherapy. Invitations were sent to individuals who scored above established cutoffs on at least one of the two anxiety subscales (*Generalized Anxiety* and *Social Anxiety*) of the Counseling Center Assessment of Psychological Symptoms [CCAPS; (58)], and who also scored below the 80th percentile for non-anxiety subscales. Formal psychiatric diagnoses are not generally given in the counseling center and thus were not available. The NT group was recruited via the psychology department research participation system and reported no history of autism spectrum diagnosis or any elevated psychiatric concern or history of diagnosis.

As shown in Table 1, the AUT group was significantly older than the ANX and NT groups. There were no significant differences in cognitive performance as measured by the Wechsler Abbreviated Scales of Intelligence – Second Edition (WASI-II). All participants who agreed to participate in this study were able to complete the auditory habituation protocol.

**Table 1:** Demographic characteristics and behavioral questionnaire responses.

	**Mean ± SD**			***F* (*df*)**	***p***	**Direction**
	**AUT**	**ANX**	**NT**		
Age	24.47 ± 6.14	21.90 ± 2.80	20.94 ± 1.72	6.62 (2.92)	0.002	AUT>ANX = NT
FSIQ	112.36 ± 10.63	112.16 ± 12.13	111.95 ± 8.21	2.32 (2.92)	0.993	AUT = ANX = NT
ASQ	27.77 ± 8.96	23.33 ± 7.18	15.61 ± 5.42	23.18 (2.92)	0.000	ASD = ANX>NT
PSWQ	50.92 ± 15.01	63.11 ± 8.75	46.69 ± 11.98	14.62 (2.86)	0.000	ANX>AUT = NT
IUS-12	28.00 ± 7.13	40.89 ± 9.56	38.96 ± 10.08	19.91 (2.85)	0.000	AUT = ANX>NT
**AASP**						
Sensitivity	44.91 ± 10.11	39.25 ± 9.90	33.22 ± 6.75	22.36 (2.77)	0.000	AUT = ANX>NT
Avoiding	48.75 ± 9.46	39.85 ± 9.49	37.19 ± 5.56	14.22 (2.77)	0.000	AUT>ANX = NT
Low Reg.	39.83 ± 7.14	32.20 ± 7.14	31.91 ± 5.85	11.82 (2.77)	0.000	AUT>ANX = NT
Seeking	52.69 ± 6.54	44.95 ± 8.80	38.67 ± 8.36	24.40 (2.77)	0.000	AUT>ANX>NT

*AUT, autistic adults; ANX, highly anxious adults; NT, neurotypical adults; FSIQ, full scale IQ from the Wechsler Abbreviated Scales of Intelligence–Second Edition; AASP, adolescent adult sensory profile; Sensory Sensitivity, Sensation Avoiding, Low Registration and Sensory Seeking subscales*.

### Behavioral Measures

#### Autism Spectrum Quotient

The Autism Spectrum Quotient [ASQ; 59] is a 50-item questionnaire that asks participants to indicate the extent to which they can identify with statements describing behaviors and attitudes that reflect core autistic traits. The ASQ has been used as a dimensional measure of autism traits in clinical populations and in the general public, and has been demonstrated to be sensitive to a range of intensity of autism symptoms (60).

#### Penn State Worry Questionnaire

The Penn State Worry Questionnaire (PSWQ) is a 16-item questionnaire that measures the severity of anxious apprehension or worry, in both clinical and nonclinical populations (61) The PSWQ has been shown to have good discriminant validity and convergent validity; to be unrelated to measures of depression (e.g., the Beck Depression Inventory) and to be sensitive to cognitive oriented treatment (61, 62).

#### Intolerance of Uncertainty Scale-12

The Intolerance of Uncertainty Scale-12 (IUS-12) (63) is a 12-item measure that includes questions about the unknown regarding one's prospective anxiety (e.g., “Unforeseen events upset me greatly”) and inhibitory anxiety (e.g., “Uncertainty keeps me from living a full life”). The IUS-12 total score was used in the current study.

#### Adolescent/Adult Sensory Profile

The Adolescent/Adult Sensory Profile [AASP; (32)] is a 60-item questionnaire measuring four sensory processing categories based on Dunn's (64) model of sensory processing: *low registration* (i.e., easily misses sensory information), *sensation seeking* (seeks out sensory stimulation), *sensory sensitivity* (heightened awareness of sensory stimuli), and *sensation avoiding* (withdraws from overwhelming sensory input). The four subscales of the AASP reflect typical sensory processing where extreme scores (higher or lower) reflect differences from typical development.

### Eye-Tracking Apparatus and Measurement

The experiment was conducted in a 6′ × 15′ room with a single window that was facing southeast. The window's blind were closed for all participants. The rooms lights consisted of four fluorescents ceiling lights that were on while the participants completed the study. Pupils were recorded via an SR Research Eyelink 1000 Plus tower mount eye tracker (spatial resolution of 0.01°) sampling at 1000 Hz. Subjects were seated 60 cm away from a 24″ LCD screen with their back toward the window to reduce effects of luminance from the outside environment. Head movements were minimized with a chin and headrest. Although viewing was binocular, recordings were taken from the right eye only. Prior to recording, the eye tracker was calibrated using a nine-point calibration routine. The experiment was controlled with SR Research Experiment Builder software.

### Auditory Habituation Protocol

After the eye-tracking equipment was calibrated to the participant, each participant was shown the instructions on the computer screen while the experimenter also read the instructions out loud. The instructions were as follows, “During this experiment, you will be staring at the fixation cross in the center of the screen. While staring at the cross you will be hearing noises in the headphones. Please keep your eyes focused on the cross throughout the experiment. Failure to look at the fixation cross will pause the experiment. Do you have any questions?” After answering any participant questions the experimenter started the protocol. The auditory habituation protocol consisted of three blocks with 10 trials per block for a total of 30 trials. The first block included only “Silence” trials consisting of a silent tone generated using Audacity software. The second block included only Sound1 trials, consisting of a 2000 Hz sinewave tone, also generated using Audacity and presented at 60 db. The last block included only Sound2 trials, consisting of a 2000 Hz sawtooth tone (which is scratchier and slightly more aversive than the sinewave tone), also generated using Audacity and presented at 80 db. Each trial began with 500 ms silence followed by the corresponding sound (Silence, Sound1, or Sound2) with a jittered duration from 1800 to 2200 ms (mean = 2000 ms). This was followed by jittered inter-trial-interval ranging from 18000 to 22000 ms (mean = 20000 s). Each participant received each block in the same order (Silence, Sound1, Sound2). During each block, the fixation-cross remained on screen continuously, and there were no visual changes to the screen to indicate that one trial had ended and another had begun. The eye-tracker was programmed so that if the eyes left a pre-defined invisible area around the fixation cross, the experiment would pause until the eyes returned to the fixation cross. Participants were instructed to stare at a black fixation cross of 200 × 200 pixels cross that was located in the center of a white screen.

### Ethical Considerations

This study was submitted to and approved by the Brigham Young University Institutional Review Board (BYU IRB). All clients were recruited in accordance to BYU IRB guidelines. In accordance with the Declaration of Helsinki, all participants signed the IRB-approved consent form that has been verbally including information that participants could withdraw from the study at any time. All data for this studied was de-identified during the data preparation phase. Participants were compensated $15 upon the completion of this study.

### Data Cleaning and Preparation

All data preparation was completed using R statistical software (65).

Because the data were originally in arbitrary area units, we converted the data to mm diameter by running the experiment with a 10 mm artificial pupil and using the resulting data to compute pupil diameter of the actual participants. Data were cleaned by manually removing samples that occurred during blinks and saccades. The data was then smoothed using a loess filter with a span of 0.25. Pupil size at time 0 (the moment before sound onset) was used as a baseline, and pupil size change was computed by subtracting this baseline value from each sample. Finally, before analysis, outlier samples greater than or less than 2.5 standard deviations from the participant's mean were removed (less than 4% of the total data were removed; the amount removed did not differ by group), and the pupil data were grouped into 250 ms bins via averaging (66).

## Results

### Aim 1: Sensory Processing in Autistic Adults

We first examined between-group differences on behavioral measures, as summarized in Table 1. The ASQ, IUS-12 total score, and AASP *sensory sensitivity* subscale had non-normal distributions and we followed standard ANOVA analyses with Kruskal-Wallis tests (with Dunn's test of multiple comparisons of rank sums using the “dunntest” package of STATA 14. The ANOVA and Kruskal-Wallis tests provided identical results in all cases.

As expected, scores for the AUT group were significantly different than the NT group on all subscales of the AASP sensory questionnaire, including higher scores on the atypical sensory experience scales and lower scores on the typical *sensory seeking* scale. ANX group scores were equivalent to the AUT group for the *sensory seeking* subscale, equivalent to the NT group for the *low registration* and *sensory avoidance* scales, and between the AUT and NT group for *sensory seeking*. The ANX group had the highest scores on the PSWQ (anxious apprehension), while the AUT and ANX groups were equivalent for the IUS-12 total (intolerance of uncertainty). In line with our previous findings regarding autism trait measures in highly anxious adults (67), the AUT and ANX groups were statistically equal for the ASQ total score.

#### Associations With Anxiety and Sensory Processing

As shown in Tables 2A, 2B, dimensional analyses of all participants combined across groups (*n* = 77) found strong significant correlations between the AASP subscales and the IUS-12 and PSWQ total scores. This is in line with our previous paper that looked at dimensional associations with autism and neurotypical groups in the same analysis (39). Breaking down the correlations by group showed a few different patterns between groups although lower statistical power due to the sample separation affects interpretation. There were no significant correlations between the pupillometry measures and any of the behavioral measures.

**Table 2A:** Association of sensory experience and intolerance of uncertainty.

	**Combined**		**AUT**		**ANX**		**NT**	
**AASP scale**	***r***	***p***	***r***	***p***	***r***	***p***	***r***	***p***
Sensory sensitivity	0.565	< 0.001	0.514	0.020	0.376	0.102	0.337	0.045
Sensory avoidant	0.570	< 0.001	0.532	0.016	0.329	0.157	0.636	< 0.001
Low registration	0.380	0.001	0.417	0.067	0.398	0.083	0.105	0.544
Sensory seeking	−0.478	< 0.001	0.147	0.536	−0.308	0.187	−0.387	0.020

**Table 2B:** Association of sensory experience and anxious apprehension.

	**Combined**		**AUT**		**ANX**		**NT**	
**AASP scale**	***r***	***p***	***r***	***p***	***r***	***p***	***r***	***p***
Sensory sensitivity	0.400	< 0.001	0.237	0.300	0.318	0.172	0.451	< 0.001
Sensory avoidant	0.271	0.017	−0.030	0.898	0.349	0.131	0.444	0.007
Low registration	0.256	0.025	0.601	0.004	0.238	0.311	0.083	0.628
Sensory seeking	−0.160	0.154	0.061	0.794	0.066	0.782	0.011	0.948

*AUT n = 20, ANX n = 21, NT n = 36. AASP, Adolescent Adult Sensory Profile; PSWQ, Penn State Worry Questionnaire; IUS-12, Intolerance of Uncertainty Scale-12*.

### Aim 2: Baseline and General Physiological Arousal

**Figure 1:**
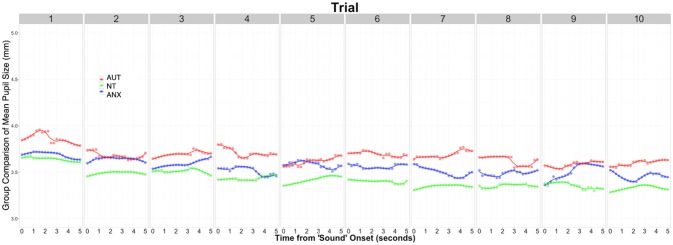
Tonic pupil size for each group across all 10 trials of the Silence condition.

We calculated the average pupil size across each of the 10 trials of the baseline Silence condition to calculate the tonic pupil size for each group as our baseline measure. Table 3 reports group differences in this measure and the following pupillometry measures. Analysis revealed significant differences between the groups [*F*(2, 92) = 3.32, *p* = 0.044]. *Post-hoc* analysis showed the AUT group had a significantly greater tonic pupil size than both the NT group and ANX groups. Figure 1 depicts this group difference in tonic pupil size on a trial-by-trial basis. Standard ANOVA analysis showed identical results. In detail, Figure 1 shows that pupil size was largest for the AUT group at the beginning of the block, with the ANX group in-between the AUT and NT groups. This pattern remained constant throughout each 5-second block of “silence” trials.

**Table 3:** Group comparisons for pupil size at various time points.

	**Mean ± SD**			***F* (*df*)**	***p***	**Direction**
	**AUT**	**ANX**	**NT**		
Baseline Pupil Size	3.92 ± 0.54	3.59 ± 0.28	3.63 ± 0.42	3.32 (2.92)	0.044	AUT = ANX>NT
Sound1 Response	0.311 ± 0.33	0.272 ± 0.32	0.298 ± 0.34	0.67 (2.92)	0.514	AUT = ANX = NT
Sound2 Response	0.715 ± 0.45	0.667 ± 0.37	0.905 ± 0.57	14.62 (2.86)	0.334	AUT = ANX = NT

*AUT, autistic adults; ANX, highly anxious adults; NT, neurotypical adults; Baseline Pupil Size, tonic pupil size averaged across all trials of the baseline “no-sound” condition. Sound1 and 2 initial responses are the difference of the peak pupil size in the 2000 ms following the first onset of that sound minus the pupil size at Time 0 immediately before the onset of that sound*.

We used Hierarchal Linear Modeling (HLM) to calculate pre-experiment pupil size and the effects of that starting point on general physiological arousal throughout the whole experiment. HLM is especially useful for these analyses because the models account for inter-individual variability with the aim to separate “true effects” from “random effects” created by individual variability (68). The AUT group's pupil size at Time 0, immediately before the Silent block began—equivalent to the intercept of the model–was significantly larger than the NT group (*t* = −2.20, *p* = 0.031), but not significantly different from the ANX group (*t* = -1.83, *p* = 0.071), indicating higher arousal of the AUT group compared to the NT group at the beginning of the experiment. Pupil size for the ANX group was in-between that of the AUT and NT groups, and secondary analysis using the ANX group as the reference showed no difference from the other two groups. Throughout the duration of the experiment, the AUT group's pupil size did not change significantly compared to the null slope of zero (*t* = 0.74, *p* = 0.428). The NT group slope was not significantly different than the AUT group (*t* = 1.86, *p* = 0.063), indicating that their mean pupil size also did not change during the course of the task. However, the ANX group showed decreased pupil size across the duration of the experiment compared to the AUT group (*t* = −2.69, *p* = 0.007) and the NT group (*t* = −2.01, *p* = 0.045). Putting these analyses together, the AUT group started with a larger pupil size than the NT group but was not significantly different than the ANX group, but the ANX group decreased over the course of the experiment while the AUT group did not, so that the difference between the two groups increased significantly over the course of the experiment.

### Aim 3: Auditory Response and Habituation

As is common with psychophysiology measurements, most pupillometry data were positively skewed and we analyzed data using Kruskal-Wallis tests with Dunn's tests for *post-hoc* comparisons. Follow-up ANOVA analyses reported identical results in every case. We divided our analyses regarding habituation into three steps. First was to compare the initial response to hearing each sound, as a measure of arousal when orienting to novel stimuli. Second was to track the rate of decline in pupil size from the offset of the sound stimulus to the beginning of the next trial. Third was to track the slope of response magnitude from trial-to-trial as a measure of habituation to each sound over the duration of the stimulus block.

### Initial Response to Sound Stimuli

We examined initial pupil response to each of the two sounds by looking at the peak pupil change within the first 2000 ms following sound onset, during the first trial for that sound. There was no between-groups difference for either sound: Sound1 [*F*(2, 92) = 0.67, *p* = 0.51], Sound2 [*F*(2, 92) = 2.29, *p* = 0.11]. Thus, there were no overall group differences in the initial response to each tone.

### Recovery After Sound Stimulus

We next analyzed potential group differences in recovery following the sound stimuli. Our first analysis showed that, across the combined Sound1 and Sound2 trials, there was no significant between-groups difference in pupil size at the time of sound offset. We then calculated the slope of pupil size for the duration from sound offset to the beginning of the next trial, using the number of seconds from sound offset as the time variable. Visual inspection of the data and unconditional growth curve models suggested that a quadratic transformation of the time (in seconds) showed the best model fit (See Supplemental Table 1). All three groups showed significant decrease in pupil size from the offset of the sound to the start of the next trial (Supplemental Table 2). The AUT group showed slower recovery than the NT group (*t* = −5.51, *p* < 0.001) and faster recovery than the ANX group (*t* = 68.65, *p* < 0.001).

### Auditory Habituation to the Sound Stimuli

Our critical question of habituation was analyzed by calculating change in per-trial pupil response across all of the sound trials. We began by calculating the difference between the baseline for each trial (i.e., mean pupil size during the 500 ms silence) and the peak pupillary response during presentation of the sound stimulus (2000 ms). We utilized HLM to model change in this response over time. Visual inspection of the data and unconditional growth models indicated that a natural log transformation of trial [“ln(trial)”] variable provided the best fit of the data for both Sounds blocks (see Supplemental Tables 3, 4). The final model for the peak change in pupil size across the Sounds blocks included fixed effects of group and trial, the group-by-trial interaction, and the random effects of trial.

**Figure 2:**
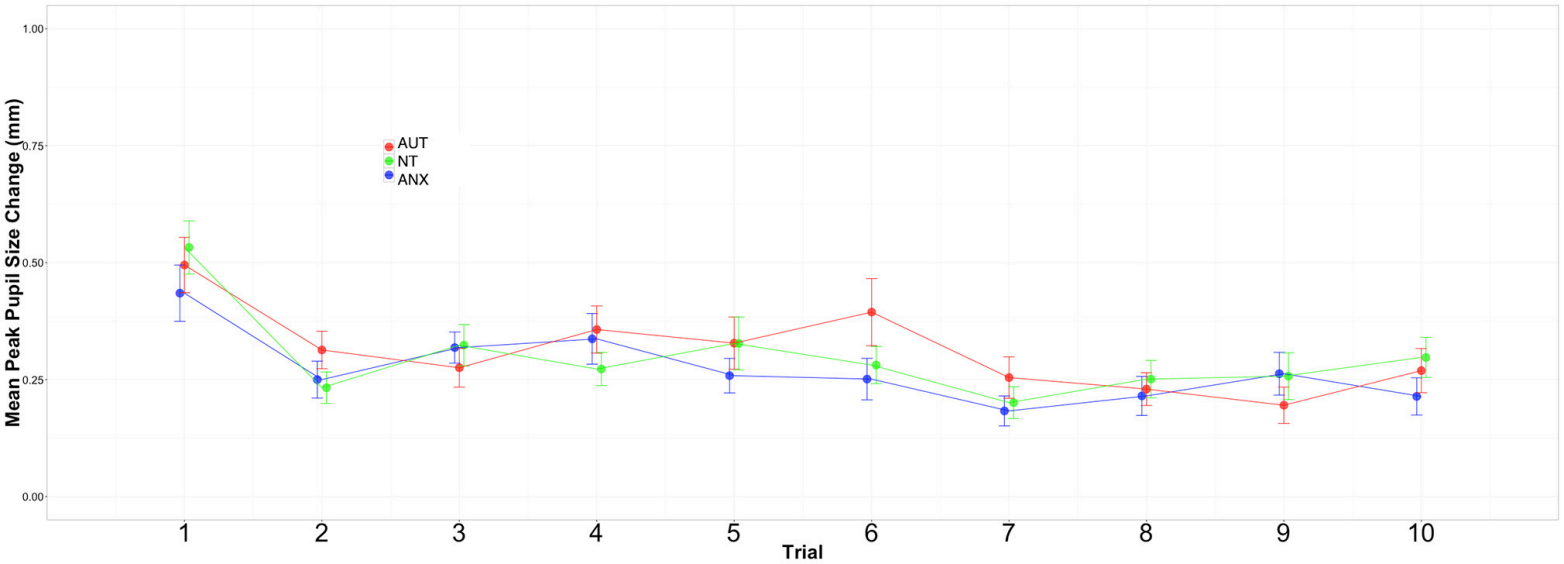
Change in peak pupil responses to Sound1 across all trials.

**Figure 3:**
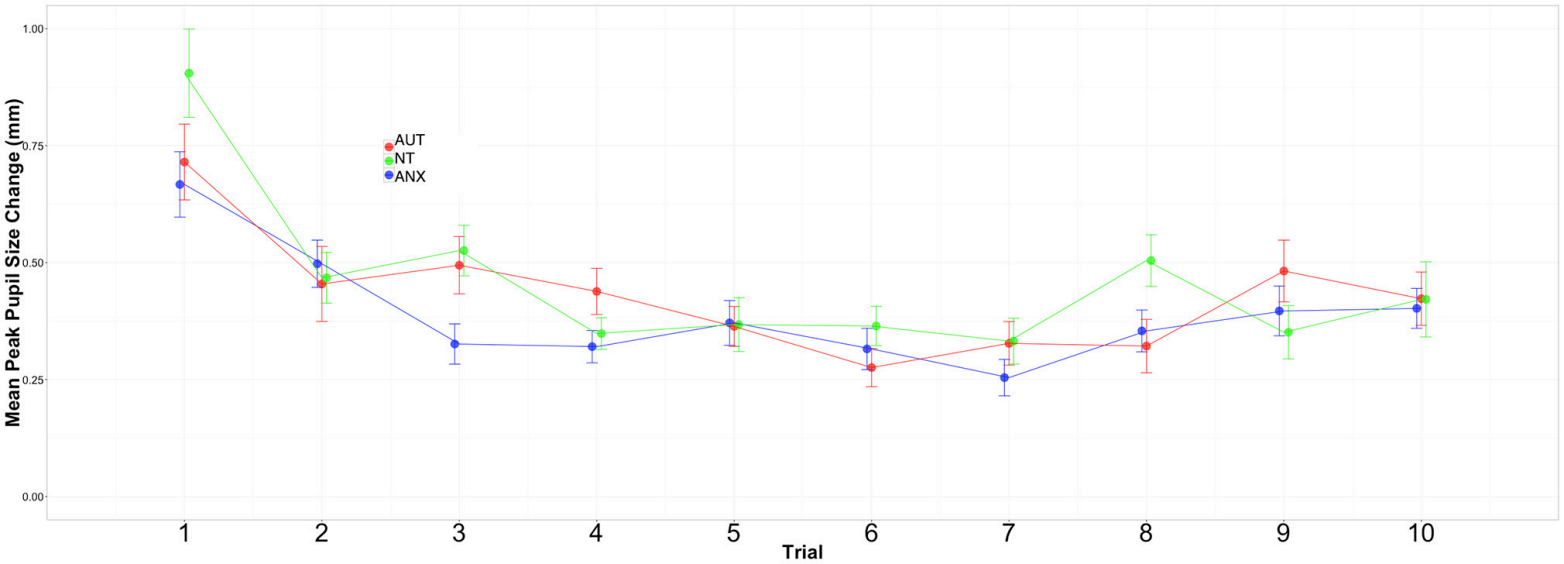
Change in peak pupil responses to Sound2 across all trials.

Figures 2, 3 depict the habituation trends. Results for the Sound 1 block showed a significant effect for trial but non-significant effects for group or the group-by-trial interaction. Thus, the three groups habituated to Sound1 at similar rates. There was likewise a strong habituation response for the Sound2 trials, but non-significant group main effects or group-by-trial interaction effects (see Supplemental Tables 5, 6).

## Discussion

There were two separate types of measures in this study: the first is tonic pupil size, that is, pupil size in the absence of any explicit sensory stimulation, which may index everyday physiological arousal that co-exists with feelings of anxiety. The second is the change in pupil size in response to sensory (i.e., auditory) stimulation which may relate to basic sensory processes. There is growing evidence to suggest that sensory sensitivity and anxiety are especially related to each other in autism (18). To our knowledge, this is the first comparison of a cross-clinical sample of autistic adults, non-autistic anxious adults and neurotypical non-anxious adults, looking directly at these measures of baseline arousal and subsequent reactivity and habituation to sensory stimuli. We will discuss the findings of this study according its three aims.

### Aim 1: Sensory Processing in Autistic Adults

The first aim of this study was to evaluate sensory processing behaviors, and their link to measures of anxious apprehension and autism traits, in autistic adults vis-à-vis clinical and non-clinical comparison groups. The Adolescent and Adult Sensory Profile (AASP) measures sensory experience extensively across four domains, and the AUT group scored higher than the NT sample in every domain, with the ANX group falling in-between. These self-report data match earlier findings from child samples that relied on parent-report surveys, and confirm that challenging sensory experiences in autism persist into adulthood. While the anxiety group did report differences from the NT sample in the domains of sensory sensitivity and sensory seeking behavior, these differences are much more pronounced in the AUT group. These findings suggest a link between anxiety and sensory experiences in which atypical sensory processing may contribute to heightened anxiety, serving as evidence that a potential mechanism for the increased in anxiety in autism is atypical sensory processing. Although the sample size in this study did not permit more sophisticated statistical modeling, our dimensional data further suggest that intolerance of uncertainty and anxiety (at least for a measure of worry/anxious apprehension) may be related to sensory processing. Further research with larger sample sizes is needed to test models of the underlying mechanisms for anxiety in autism similar to studies such as those done with alexithymia (31, 39).

### Aim 2: Baseline Arousal and General Physiological Arousal in Autistic Adults

A notable finding from our pupillometry measures is increased pupil dilation in the ASD group at baseline (before any auditory stimulation). In the absence of a known physical reason for between-group differences in pupil size, it is possible that this difference reflects ongoing elevated physiological arousal in the autism sample. As reviewed in Aim 3 below, the measure of auditory habituation showed that the autism group habituated to the stimuli at similar rates as the comparison groups. However, larger absolute pupil size persisted in the autism group throughout the course of the experiment. That is, even as pupil size decreased with habituation to the sound stimulus for all groups, pupil size in the AUT group never came down to match the other groups. This elevation may reflect frequently increased activation of the sympathetic nervous system in autism that does not diminish over time. Such chronic hyperarousal could function as a mechanism and/or consequence of anxiety in autism. While not a universal finding in the autism literature, there are an increasing number of suggestions that everyday physiological arousal is atypical in many autistic people and could include chronic physiological arousal (35, 42, 69).

This idea was also suggested by the first ever fMRI study of fear conditioning in autism (43), where we found much stronger amygdala activation to threat vs. safe cues in a neurotypical adult sample, but significantly reduced differentiation between threat and safe in the autistic sample. We wondered whether elevated baseline arousal—either in everyday life and/or as a function of the intense sensory environment of the MRI setting—provided a sort of ceiling effect for amygdala activation so that additional, task-based activation was less likely. We have recently undertaken a study of physiolgical arousal over the course of a psychotherapy session which may shed light on this. Studies with ambulatory or other, more ecologically valid approaches would certiainly be useful for elucidating these possibilities.

### Aim 3: Auditory Response and Habituation

Habituation is defined as an exponential decrement of a response to an initially novel stimulus that is presented repeatedly over time (55, 70). Both animal and human research provide strong support for links between less successful habituation with less flexible adaptation that may underlie anxiety. One intriguing model of autism (71, 72) suggests that challenges integrating prior and current environmental input—for example difficulties with sensory habituation—could drive a unique sensory-perceptual experience that can make the autistic world seem “too real” and overwhelming. However, our study found no group differences in the initial response to sound stimuli (i.e., pupil size change when hearing the first sound) or in habituation to those stimuli over time. Thus, our data do not support a link between atypical sensory habituation and anxiety. However, this may be because ours was a very simple task that required no activity or active learning. It may be that increased task demands could over-tax sensory integration systems, as suggested by fMRI studies from Green et al. (52, 53) who sowed atypical sensory response only for simultaneous stimulation of multiple sensory systems, and not for single sensory modalities presented separately. Possible questions for future research include: Could it be that chronically elevated physiological arousal, and/or an elevated response to lab-based stressors, could modulate findings in habituation studies (43)? Is habituation decreased for unimodal sensory stimulation (i.e., only auditory or only tactile) or do difficulties appear only in the integration of multiple stimuli at once (as is common in real life) (52)? Does uncertainty associated with sensory processing challenges directly contribute to the intolerance of uncertainty that seems so prominent in autism (18, 20)?

### Limitations

There are several limitations for this study. Firstly, the compositions of our groups were different on multiple levels. For instance, the ASD group was significantly older than the ANX and NT groups. However, HLM analyses indicated that age was not a significant predictor of pupil responses. There were more females in the ANX group than in the ASD or NT groups. Some research has shown that females have larger pupil responses than males to neutral stimuli (73). The ANX group was not formally diagnosed with anxiety disorders, and we did not assume a formal diagnosis in our conceptual or experimental findings. They were a group of individuals (a) who were actively seeking treatment for emotional distress; (b) who scored high on common intake measures of anxiety used widely in college counseling centers, and not so high on depression; (c) scored high on study measures of anxious apprehension/worry (the PSWQ) as well as on the intolerance of uncertainty measure (IUS-12). But subsequent studies with carefully characterized clinical groups including anxiety are necessary before making any stronger conclusions about the overlap of anxiety and autism. As noted above, the limited sample size precluded mediation modeling and other useful approaches. The lack of correlation between our self-report questionnaires and observed psychophysiological responses is predicted by recent arguments from LeDoux et al. that psychophysiological defense mechanisms are separate from the subjective, conscious experience of fear (74, 75) although this framework is quite controversial. Linking psychophysiology with questionnaire data has been traditionally problematic in autism (76) and more research about how different systems might feed into each other is an important and ripe area for research.

This study also has some strength. We believe that the involvement of additional clinical samples such as anxiety is an essential approach for research moving forward, as is now happening in many research groups. Pupillometry is a simple and non-invasive physiological measure that precluded participant attrition. The auditory habituation task was the simplest possible protocol to test our hypotheses, examining basic sensory processes that are less reliant on higher-level cognitive processes.

### Clinical Implications

While many autistic adults figure out how to compensate for differences in social styles and motivation, and could find success in relationships, employment and other settings, success is often impeded by overwhelming feelings of anxiety. Many autistic adults continue to be bothered by sensory stimulation that is disruptive in its own right and may further exacerbate anxiety. One autistic adult in our study reported that he feels “at war with the world” because of frequently overwhelming sensory stimulation. This can lead to frequent feelings of confusion and uncertainty that mediate the link between sensory experience and anxiety, and could contribute to everyday feelings of challenge and heightened physiological arousal.

Attention to sensory experience is not a standard element of cognitive behavioral therapy (CBT) or other treatment modalities. In light of increased awareness of how sensory experience and anxiety uniquely interact in autism, an explicit focus on sensory processing challenges will likely be beneficial for many children and adults in home, school, work, and therapeutic settings (18, 77–79). Consultation with the autistic student/employee/client and those who know them well can be essential for understanding the nature of sensory and anxiety experiences and learning how to utilize that information to build supports and/or interventions to alleviate sensory challenges (80, 81). Such approaches could include additional environmental supports or changes (including those used in occupational therapy), as well the autistic person learning how to manage sensory challenges more effectively.

As understanding grows of cognitive, emotional, and sensory contributions to anxiety in autism, it is imperative to assimilate targeted treatment approaches—certainly behavioral and possibly pharmacological approaches—into autism interventions (9, 36, 39, 79, 82, 83). Anxiety in autism *is different* than anxiety without autism, and intervention approaches need to adapt accordingly. At the same time, it is essential to further explore heterogeneity in autism. Sensory experience, and anxiety experience (and alexithymia and intolerance of uncertainty and many other constructs) are not universal within autism. Several recent studies have highlighted the importance of examining varying levels of anxiety within large autism samples (48, 84).

Consulting with the autistic person on what challenges are most detrimental to their success is essential. Understanding that typical approaches to anxiety have considerable efficacy in autism is helpful [e.g., (85–89)]. But it is equally necessary to realize that there are important, unique aspects of anxiety in autism including (a) differences in central and autonomic nervous system function (15, 48, 84); the validity of typical anxiety symptom questionnaires (90–92); and helpful modifications for treatment (80, 81, 93). Thus, behavioral and pharmacological treatments for anxiety in autism should think outside the box, including explicit and dedicated attention to the impact of atypical sensory experience in so many autistic children and adults.

## Author Contributions

DT, SL, and MS conceptualized and designed the project. DT and KS were involved in data collection with equipment provided by SL. DT, KS, and SL completed data processing and all authors contributed to statistical analysis. DT and MS wrote the manuscript which was reviewed and approved by all authors.

### Conflict of Interest Statement

The authors declare that the research was conducted in the absence of any commercial or financial relationships that could be construed as a potential conflict of interest.

## References

[1] Tomchek SD Dunn W. Sensory processing in children with and without autism: a comparative study using the short sensory profile. Am J Occup Ther Off Publ Am Occup Ther Assoc. (2007) 61:190–200. 10.5014/ajot.61.2.190 17436841

[2] Tomchek SD Huebner RA Dunn W Patterns of sensory processing in children with an autism spectrum disorder. Res Autism Spectr Disord. (2014) 8:1214–24. 10.1016/j.rasd.2014.06.006

[3] Baranek GT Boyd BA Poe MD David FJ Watson LR MacLean WE Jr. Hyperresponsive sensory patterns in young children with autism, developmental delay, and typical development. Am J Ment Retard. (2007) 112:233–45. 10.1352/0895-8017(2007)112[233:HSPIYC]2.0.CO;2 17559291

[4] Green D Chandler S Charman T Simonoff E Baird G. Brief report: DSM-5 sensory behaviours in children with and without an autism spectrum disorder. J Autism Dev Disord. (2016) 46:3597–606. 10.1007/s10803-016-2881-7 27475418

[5] Leekam SR Nieto C Libby SJ Wing L Gould J. Describing the sensory abnormalities of children and adults with autism. J Autism Dev Disord. (2006) 37:894–910. 10.1007/s10803-006-0218-7 17016677

[6] American Psychiatric Association Diagnostic and Statistical Manual of Mental Disorders, 5th Edition. DSM-V. American Psychiatric Association Publishing (2013).

[7] Christensen DL Baio J Braun KVN Bilder D Charles J Constantino JN Prevalence and characteristics of autism spectrum disorder among children aged 8 years — autism and developmental disabilities monitoring network, 11 Sites, United States, 2012. MMWR Surveill Summ. (2016) 65:1–23. 10.15585/mmwr.ss6503a1 PMC7909709 27031587

[8] Buck TR Viskochil J Farley M Coon H McMahon WM Morgan J. Psychiatric comorbidity and medication use in adults with autism spectrum disorder. J Autism Dev Disord. (2014) 44:3063–71. 10.1007/s10803-014-2170-2 24958436 PMC4355011

[9] Kerns CM Kendall PC Berry L Souders MC Franklin ME Schultz RT. Traditional and atypical presentations of anxiety in youth with autism spectrum disorder. J Autism Dev Disord. (2014) 44:2851–61. 10.1007/s10803-014-2141-7 24902932 PMC5441227

[10] van Steensel FJA Bögels SM de Bruin EI Psychiatric comorbidity in children with autism spectrum disorders: a comparison with children with ADHD. J Child Fam Stud. (2013) 22:368–76. 10.1007/s10826-012-9587-z 23524401 PMC3602612

[11] Gotham K Bishop SL Hus V Huerta M Lund S Buja A. Exploring the relationship between anxiety and insistence on sameness in autism spectrum disorders. Autism Res Off J Int Soc Autism Res. (2013) 6:33–41. 10.1002/aur.1263 23258569 PMC4373663

[12] Rodgers J Glod M Connolly B McConachie H. The relationship between anxiety and repetitive behaviours in autism spectrum disorder. J Autism Dev Disord. (2012) 42:2404–9. 10.1007/s10803-012-1531-y 22527704

[13] Luke L Clare ICH Ring H Redley M Watson P. Decision-making difficulties experienced by adults with autism spectrum conditions. Autism Int J Res Pract. (2012) 16:612–21. 10.1177/1362361311415876 21846664

[14] Conner CM Maddox BB White SW. Parents' state and trait anxiety: relationships with anxiety severity and treatment response in adolescents with autism spectrum disorders. J Autism Dev Disord. (2013) 43:1811–8. 10.1007/s10803-012-1728-0 23224592 PMC11097144

[15] Corbett BA Muscatello RA Blain SD. Impact of sensory sensitivity on physiological stress response and novel peer interaction in children with and without autism spectrum disorder. Front Neurosci. (2016) 10:278. 10.3389/fnins.2016.00278 27445653 PMC4917546

[16] Green SA Ben-Sasson A. Anxiety disorders and sensory over-responsivity in children with autism spectrum disorders: is there a causal relationship? J Autism Dev Disord. (2010) 40:1495–504. 10.1007/s10803-010-1007-x 20383658 PMC2980623

[17] Lidstone J Uljarević M Sullivan J Rodgers J McConachie H Freeston M Relations among restricted and repetitive behaviors, anxiety and sensory features in children with autism spectrum disorders. Res Autism Spectr Disord. (2014) 8:82–92. 10.1016/j.rasd.2013.10.001

[18] South M Rodgers J. Sensory, emotional and cognitive contributions to anxiety in autism spectrum disorders. Front Hum Neurosci. (2017) 11:20. 10.3389/fnhum.2017.00020 28174531 PMC5258728

[19] Uljarević M Carrington S Leekam S. Brief report: effects of sensory sensitivity and intolerance of uncertainty on anxiety in mothers of children with autism spectrum disorder. J Autism Dev Disord. (2015) 46:315–9. 10.1007/s10803-015-2557-8 26254895

[20] Wigham S Rodgers J South M McConachie H Freeston M. The interplay between sensory processing abnormalities, intolerance of uncertainty, anxiety and restricted and repetitive behaviours in autism spectrum disorder. J Autism Dev Disord. (2015) 45:943–52. 10.1007/s10803-014-2248-x 25261248

[21] Gasquoine PG. Contributions of the insula to cognition and emotion. Neuropsychol Rev. (2014) 24:77–87. 10.1007/s11065-014-9246-9 24442602

[22] Lübke KT Pause BM. Always follow your nose: the functional significance of social chemosignals in human reproduction and survival. Horm Behav. (2015) 68:134–44. 10.1016/j.yhbeh.2014.10.001 25637403

[23] Soumiya H Godai A Araiso H Mori S Furukawa S Fukumitsu H. Neonatal whisker trimming impairs fear/anxiety-related emotional systems of the amygdala and social behaviors in adult mice. PLoS ONE (2016) 11:e0158583. 10.1371/journal.pone.0158583 27362655 PMC4928826

[24] Acevedo BP Jagiellowicz J Aron E Marhenke R Aron A Acevedo B Sensory processing sensitivity and childhood quality's effects on neural responses to emotional stimuli. Clin Neuropsychiatry (2017) 14:359–73.

[25] Ahadi B Basharpoor S Relationship between sensory processing sensitivity, personality dimensions and mental health. J Appl Sci. (2010) 10:570–4. 10.3923/jas.2010.570.574

[26] Aron EN Aron A. Sensory-processing sensitivity and its relation to introversion and emotionality. J Pers Soc Psychol. (1997) 73:345–68. 9248053 10.1037//0022-3514.73.2.345

[27] Wallis K Sutton D Bassett S Sensory modulation for people with anxiety in a community mental health setting. Occup Ther Ment Health (2018) 34:122–37. 10.1080/0164212X.2017.1363681

[28] Neil L Olsson NC Pellicano E. The relationship between intolerance of uncertainty, sensory sensitivities, and anxiety in autistic and typically developing children. J Autism Dev Disord. (2016) 46:1962–73. 10.1007/s10803-016-2721-9 26864157 PMC4860201

[29] Green SA Ben-Sasson A Soto TW Carter AS. Anxiety and sensory over-responsivity in toddlers with autism spectrum disorders: Bidirectional effects across time. J Autism Dev Disord. (2012) 42:1112–9. 10.1007/s10803-011-1361-3 21935727 PMC4199633

[30] Uljarević M Prior MR Leekam SR. First evidence of sensory atypicality in mothers of children with Autism Spectrum Disorder (ASD). Mol Autism (2014) 5:26. 10.1186/2040-2392-5-26 24694290 PMC3975853

[31] Milosavljevic B Carter Leno V Simonoff E Baird G Pickles A Jones CRG. Alexithymia in adolescents with autism spectrum disorder: its relationship to internalising difficulties, sensory modulation and social cognition. J Autism Dev Disord. (2016) 46:1354–67. 10.1007/s10803-015-2670-8 26659552

[32] Brown C Tollefson N Dunn W Cromwell R Filion D. The adult sensory profile: measuring patterns of sensory processing. Am J Occup Ther. (2001) 55:75–82. 10.5014/ajot.55.1.75 11216370

[33] Dugas MJ Gagnon F Ladouceur R Freeston MH. Generalized anxiety disorder: a preliminary test of a conceptual model. Behav Res Ther. (1998) 36:215–26. 10.1016/S0005-7967(97)00070-3 9613027

[34] Boulter C Freeston M South M Rodgers J. Intolerance of uncertainty as a framework for understanding anxiety in children and adolescents with autism spectrum disorders. J Autism Dev Disord. (2014) 44:1391–402. 10.1007/s10803-013-2001-x 24272526

[35] Chamberlain PD Rodgers J Crowley MJ White SE Freeston MH South M. A potentiated startle study of uncertainty and contextual anxiety in adolescents diagnosed with autism spectrum disorder. Mol Autism (2013) 4:31. 10.1186/2040-2392-4-31 24007557 PMC3844321

[36] Keefer A Kreiser NL Singh V Blakeley-Smith A Duncan A Johnson C. Intolerance of uncertainty predicts anxiety outcomes following CBT in youth with ASD. J Autism Dev Disord. (2016) 47:3949–58 10.1007/s10803-016-2852-z 27405445

[37] Einstein DA. Extension of the transdiagnostic model to focus on intolerance of uncertainty: a review of the literature and implications for treatment. Clin Psychol Sci Pract. (2014) 21:280–300. 10.1111/cpsp.12077 25400336 PMC4204511

[38] McEvoy PM Mahoney AEJ. To be sure, to be sure: intolerance of uncertainty mediates symptoms of various anxiety disorders and depression. Behav Ther. (2012) 43:533–45. 10.1016/j.beth.2011.02.007 22697442

[39] Maisel ME Stephenson KG South M Rodgers J Freeston MH Gaigg SB. Modeling the cognitive mechanisms linking autism symptoms and anxiety in adults. J Abnorm Psychol. (2016) 125:692–703. 10.1037/abn0000168 27196436

[40] Ben-Sasson A Hen L Fluss R Cermak SA Engel-Yeger B Gal E. A meta-analysis of sensory modulation symptoms in individuals with autism spectrum disorders. J Autism Dev Disord. (2008) 39:1–11. 10.1007/s10803-008-0593-3 18512135

[41] Liss M Mailloux J Erchull MJ The relationships between sensory processing sensitivity, alexithymia, autism, depression, and anxiety. Person Individ Differ. (2008) 45:255–9. 10.1016/j.paid.2008.04.009

[42] Takahashi H Komatsu S Nakahachi T Ogino K Kamio Y. Relationship of the acoustic startle response and its modulation to emotional and behavioral problems in typical development children and those with autism spectrum disorders. J Autism Dev Disord. (2016) 46:534–43. 10.1007/s10803-015-2593-4 26362152

[43] Top Jr DN Stephenson KG Doxey CR Crowley MJ Kirwan CB South M. Atypical amygdala response to fear conditioning in autism spectrum disorder. Biol Psychiatry Cogn Neurosci Neuroimaging (2016) 1:308–15. 10.1016/j.bpsc.2016.01.008 29560865

[44] Anderson CJ Colombo J. Larger tonic pupil size in young children with autism spectrum disorder. Dev Psychobiol. (2009) 51:207–11. 10.1002/dev.20352 18988196 PMC3744086

[45] Anderson CJ Colombo J Unruh KE. Pupil and salivary indicators of autonomic dysfunction in autism spectrum disorder. Dev Psychobiol. (2013) 55:465–82. 10.1002/dev.21051 22644965 PMC3832142

[46] Nuske HJ Vivanti G Dissanayake C. No evidence of emotional dysregulation or aversion to mutual gaze in preschoolers with autism spectrum disorder: an eye-tracking pupillometry study. J Autism Dev Disord. (2015) 45:3433–45 10.1007/s10803-015-2479-5 26031923

[47] Martineau J Hernandez N Hiebel L Roché L Metzger A Bonnet-Brilhault F. Can pupil size and pupil responses during visual scanning contribute to the diagnosis of autism spectrum disorder in children? J Psychiatr Res. (2011) 45:1077–82. 10.1016/j.jpsychires.2011.01.008 21679969

[48] Herrington JD Maddox BB Kerns CM Rump K Worley JA Bush JC. Amygdala volume differences in autism spectrum disorder are related to anxiety. J Autism Dev Disord. (2017) 47:3682–91. 10.1007/s10803-017-3206-1 28689329

[49] Uddin LQ. Salience processing and insular cortical function and dysfunction. Nat Rev Neurosci. (2015) 16:55–61. 10.1038/nrn3857 25406711

[50] Uddin LQ Menon V. The anterior insula in autism: under-connected and under-examined. Neurosci Biobehav Rev. (2009) 33:1198–203. 10.1016/j.neubiorev.2009.06.002 19538989 PMC2743776

[51] Herry C Bach DR Esposito F Salle FD Perrig WJ Scheffler K. Processing of temporal unpredictability in human and animal amygdala. J Neurosci. (2007) 27:5958–66. 10.1523/JNEUROSCI.5218-06.2007 17537966 PMC6672268

[52] Green SA Hernandez L Tottenham N Krasileva K Bookheimer SY Dapretto M. Neurobiology of sensory overresponsivity in youth with autism spectrum disorders. JAMA Psychiatry (2015) 72:778–86. 10.1001/jamapsychiatry.2015.0737 26061819 PMC4861140

[53] Green SA Rudie JD Colich NL Wood JJ Shirinyan D Hernandez L. Overreactive brain responses to sensory stimuli in youth with autism spectrum disorders. J Am Acad Child Adolesc Psychiatry (2013) 52:1158–72. 10.1016/j.jaac.2013.08.004 24157390 PMC3820504

[54] Järvinen A Ng R Crivelli D Neumann D Arnold AJ Woo-VonHoogenstyn N. Social functioning and autonomic nervous system sensitivity across vocal and musical emotion in williams syndrome and autism spectrum disorder. Dev Psychobiol. (2015) 58:17–26. 10.1002/dev.21335 26248474 PMC6462219

[55] Madsen GF Bilenberg N Cantio C Oranje B. Increased prepulse inhibition and sensitization of the startle reflex in autistic children. Autism Res. (2014) 7:94–103. 10.1002/aur.1337 24124111

[56] Puts NAJ Wodka EL Tommerdahl M Mostofsky SH Edden RAE. Impaired tactile processing in children with autism spectrum disorder. J Neurophysiol. (2014) 111:1803–11. 10.1152/jn.00890.2013 24523518 PMC4044368

[57] Lord C Risi S Lambrecht L Cook EH Leventhal BL DiLavore PC. The autism diagnostic observation schedule-generic: a standard measure of social and communication deficits associated with the spectrum of autism. J Autism Dev Disord. (2000) 30:205–23. 10.1023/A:1005592401947 11055457

[58] Locke BD Buzolitz JS Lei P-W Boswell JF McAleavey AA Sevig TD. Development of the counseling center assessment of psychological symptoms-62 (CCAPS-62). J Couns Psychol. (2011) 58:97–109. 10.1037/a0021282 21133541

[59] Baron-Cohen S Wheelwright S Skinner R Martin J Clubley E. The autism-spectrum quotient (AQ): evidence from Asperger syndrome/high-functioning autism, males and females, scientists and mathematicians. J Autism Dev Disord. (2001) 31:5–17. 10.1023/A:1005653411471 11439754

[60] Bishop DVM Maybery M Maley A Wong D Hill W Hallmayer J. Using self-report to identify the broad phenotype in parents of children with autistic spectrum disorders: a study using the Autism-Spectrum Quotient. J Child Psychol Psychiatry (2004) 45:1431–6. 10.1111/j.1469-7610.2004.00849.x 15482503

[61] Meyer TJ Miller ML Metzger RL Borkovec TD. Development and validation of the Penn State Worry Questionnaire. Behav Res Ther. (1990) 28:487–95. 2076086 10.1016/0005-7967(90)90135-6

[62] Dear BF Titov N Sunderland M McMillan D Anderson T Lorian C. Psychometric comparison of the generalized anxiety disorder scale-7 and the penn state worry questionnaire for measuring response during treatment of generalised anxiety disorder. Cogn Behav Ther. (2011) 40:216–27. 10.1080/16506073.2011.582138 21770844

[63] Carleton RN Norton MAPJ Asmundson GJG. Fearing the unknown: A short version of the Intolerance of Uncertainty Scale. J Anxiety Disord. (2007) 21:105–17. 10.1016/j.janxdis.2006.03.014 16647833

[64] Dunn W The impact of sensory processing abilities on the daily lives of young children and their families: a conceptual model. Infants Young Child (1997) 9:23–35.

[65] R Core Team. R: A Language and Environment for Statistical Computing. Vienna: R Foundation for Statistical Computing (2013). Available online at: http://www.R-project.org/

[66] Sirois S Brisson J. Pupillometry. Wiley Interdiscip Rev Cogn Sci. (2014) 5:679–92. 10.1002/wcs.1323 26308873

[67] South M Carr AW Stephenson KG Maisel ME Cox JC. Symptom overlap on the srs-2 adult self-report between adults with asd and adults with high anxiety. Autism Res Off J Int Soc Autism Res. (2017) 10:1215–20. 10.1002/aur.1764 28266790

[68] Singer JD Willett JB Willett CWEPJB Willett JB Applied Longitudinal Data Analysis: Modeling Change and Event Occurrence. Oxford, UK: Oxford University Press (2003).

[69] Chiu TA Anagnostou E Brian J Chau T Kushki A. Specificity of autonomic arousal to anxiety in children with autism spectrum disorder. Autism Res. (2016) 9:491–501. 10.1002/aur.1528 26389543

[70] Sinclair D Oranje B Razak KA Siegel SJ Schmid S. Sensory processing in autism spectrum disorders and Fragile X syndrome-from the clinic to animal models. Neurosci Biobehav Rev. (2016) 76:235–53. 10.1016/j.neubiorev.2016.05.029 27235081 PMC5465967

[71] Pellicano E Burr D When the world becomes “too real”: a Bayesian explanation of autistic perception. Trends Cogn Sci. (2012) 16:504–10. 10.1016/j.tics.2012.08.009 22959875

[72] Lawson RP Rees G Friston KJ. An aberrant precision account of autism. Front Hum Neurosci. (2014) 8:302. 10.3389/fnhum.2014.00302 24860482 PMC4030191

[73] Partala T Surakka V Pupil size variation as an indication of affective processing. Int J Hum Comput Stud. (2003) 59:185–98. 10.1016/S1071-5819(03)00017-X

[74] LeDoux JE Brown R. A higher-order theory of emotional consciousness. Proc Natl Acad Sci USA. (2017) 114:E2016–25. 10.1073/pnas.1619316114 28202735 PMC5347624

[75] LeDoux JE Pine DS. Using neuroscience to help understand fear and anxiety: a two-system framework. Am J Psychiatry (2016) 173:1083–93. 10.1176/appi.ajp.2016.16030353 27609244

[76] Geurts HM Corbett B Solomon M. The paradox of cognitive flexibility in autism. Trends Cogn Sci. (2009) 13:74–82. 10.1016/j.tics.2008.11.006 19138551 PMC5538880

[77] Howe FEJ Stagg SD How sensory experiences affect adolescents with an autistic spectrum condition within the classroom. J Autism Dev Disord. (2016) 46:1656–68. 10.1007/s10803-015-2693-1 26791372 PMC4826419

[78] Ozsivadjian A Knott F. Anxiety problems in young people with autism spectrum disorder: a case series. Clin Child Psychol Psychiatry (2011) 16:203–14. 10.1177/1359104511404749 21571763

[79] van Steensel FJA Heeman EJ Anxiety levels in children with autism spectrum disorder: a meta-analysis. J Child Fam Stud. (2017) 26:1753–67. 10.1007/s10826-017-0687-7 28680259 PMC5487760

[80] Rodgers J Hodgson A Shields K Wright C Honey E Freeston M. Towards a treatment for intolerance of uncertainty in young people with autism spectrum disorder: development of the coping with uncertainty in everyday situations (CUES©) programme. J Autism Dev Disord. (2016) 47:3959–66. 10.1007/s10803-016-2924-0 27796728 PMC5676830

[81] Rodgers J Ofield A. Understanding, recognising and treating co-occurring anxiety in autism. Curr Dev Disord Rep. (2018) 5:58–64. 10.1007/s40474-018-0132-7 29497597 PMC5818555

[82] Vasa RA Keefer A Reaven J South M White SW. Priorities for advancing research on youth with autism spectrum disorder and co-occurring anxiety. J Autism Dev Disord. (2017) 48:925–34. 10.1007/s10803-017-3320-0 29164436

[83] White SW Mazefsky CA Dichter GS Chiu PH Richey JA Ollendick TH. Social-cognitive, physiological, and neural mechanisms underlying emotion regulation impairments: understanding anxiety in autism spectrum disorder. Int J Dev Neurosci Off J Int Soc Dev Neurosci. (2014) 39:22–36. 10.1016/j.ijdevneu.2014.05.012 24951837 PMC4180783

[84] Hollocks MJ Howlin P Papadopoulos AS Khondoker M Simonoff E. Differences in HPA-axis and heart rate responsiveness to psychosocial stress in children with autism spectrum disorders with and without co-morbid anxiety. Psychoneuroendocrinology (2014) 46:32–45. 10.1016/j.psyneuen.2014.04.004 24882156

[85] Reaven J Blakeley-Smith A Leuthe E Moody E Hepburn S. Facing your fears in adolescence: cognitive-behavioral therapy for high-functioning autism spectrum disorders and anxiety. Autism Res Treat. (2012) 2012:423905. 10.1155/2012/423905 23091719 PMC3471403

[86] Storch EA Arnold EB Lewin AB Nadeau JM Jones AM De Nadai AS. The effect of cognitive-behavioral therapy versus treatment as usual for anxiety in children with autism spectrum disorders: a randomized, controlled trial. J Am Acad Child Adolesc Psychiatry (2013) 52:132–142.e2. 10.1016/j.jaac.2012.11.007 23357440

[87] van Steensel FJA Bögels SM CBT for anxiety disorders in children with and without autism spectrum disorders. J Consult Clin Psychol. (2015) 83:512–23. 10.1037/a0039108 25894668

[88] Wood JJ Drahota A Sze K Har K Chiu A Langer DA. Cognitive behavioral therapy for anxiety in children with autism spectrum disorders: a randomized, controlled trial. J Child Psychol Psychiatry (2009) 50:224–234. 10.1111/j.1469-7610.2008.01948.x 19309326 PMC4231198

[89] Wood JJ Ehrenreich-May J Alessandri M Fujii C Renno P Laugeson E. Cognitive behavioral therapy for early adolescents with autism spectrum disorders and clinical anxiety: a randomized, controlled trial. Behav Ther. (2015) 46:7–19. 10.1016/j.beth.2014.01.002 25526831 PMC4272761

[90] Glod M Creswell C Waite P Jamieson R McConachie H Don South M. Comparisons of the factor structure and measurement invariance of the spence children's anxiety scale-parent version in children with autism spectrum disorder and typically developing anxious children. J Autism Dev Disord. (2017) 47:3834–46. 10.1007/s10803-017-3118-0 28393292 PMC5676838

[91] Magiati I Lerh JW Hollocks MJ Uljarevic M Rodgers J McConachie H. The measurement properties of the spence children's anxiety scale-parent version in a large international pooled sample of young people with autism spectrum disorder. Autism Res. (2017) 10:1629–52. 10.1002/aur.1809 28574646

[92] Rodgers J Wigham S McConachie H Freeston M Honey E Parr JR. Development of the anxiety scale for children with autism spectrum disorder (asc-asd). Autism Res. (2016) 9:1205–15. 10.1002/aur.1603 26887910

[93] Kerns CM Roux AM Connell JE Shattuck PT Adapting cognitive behavioral techniques to address anxiety and depression in cognitively able emerging adults on the autism spectrum. Cogn Behav Pract. (2016) 23:329–40. 10.1016/j.cbpra.2016.06.002

