# Supplementary material for: Psychophysiological Arousal and Auditory Sensitivity in a Cross-Clinical Sample of Autistic and Non-autistic Anxious Adults
Source: Front Psychiatry. 2019 Jan 29;9:783. doi: 10.3389/fpsyt.2018.00783 (PMC6361859; doi:10.3389/fpsyt.2018.00783)
Supplement: Supplementary file 1 [file Data_Sheet_1.PDF]

Supplemental Table 1.

Unconditional Growth Model Fit Indices for Recovery Analysis

Variable	AIC	BIC
Time	5200.56	5235.91
ln(time)	5087.32	5106.87
Poly(time)	5023.45	5055.31

Supplemental Table 2

HLM Results of Recovery Period after Sound Offset, with Quadratic Transformation of Time

Variable	Coefficient	Std. Error	t-value	*p*-value
Intercept (ASD)	.085	.0601	1.41	.159
Group (CON)	.055	.0820	0.67	.500
Group (ANX)	-.024	.0873	-0.28	.784
Poly(Time) (ASD)	-277.921	1.263	-220.12	.000
Group (CON) * Poly(Time)	-9.577	1.737	-5.51	.000
Group(ANX) * Poly(Time)	123.849	1.804	68.65	.000

Note: ASD group is serving as the reference group.

Supplemental Table 3

Unconditional Growth Model Fit Indices for Sound1

Variable	AIC	BIC
Trial	118.71	157.54
ln(Trial)	99.93	138.76
Poly(Trial)	110.36	150.74

Supplemental Table 4

Unconditional Growth Model Fit Indices for Sound2

Variable	AIC	BIC
Trial	565.68	604.47
ln(Trial)	505.14	544.93
Poly(Trial)	521.66	5562.19

Supplemental Table 5

HLM Results Sound1 With Natural Log Transformation of Time

Variable	Coefficient	Std. Error	z-value	*p*-value
Group	.025	.0313	.83	.407
ln(Trial)	-.084	.013	-6.26	.000
Group* ln(Trial)	.004	.0171	-.26	.794
Intercept	.421	.0246	17.07	.000

Supplemental Table 6

HLM Results Sound2 With Natural Log Transformation of Time

Variable	Coefficient	Std. Error	z-value	*p*-value
Group	.023	.0426	0.52	.606
ln(Trial)	-.142	.0182	-7.79	.000
Group* ln(Trial)	-.003	.0231	-0.15	.880
Intercept	.644	.0363	17.75	.000
